# Acute restraint stress modifies the heart rate biorhythm in the poststress period

**DOI:** 10.1038/s41598-019-38523-9

**Published:** 2019-02-11

**Authors:** Eva Varejkova, Katerina Janisova, Jaromir Myslivecek

**Affiliations:** Institute of Physiology, 1st Faculty of Medicine, Charles University, Prague, Czech Republic

## Abstract

We studied the changes in the heart and the activity biorhythms in mice exposed to acute (one 120-minute session) and repeated (7 two-hour sessions) restraint stress in 129J1/CF1 mice (WT) and in mice without M_2_ muscarinic receptors (M_2_KO) during the prestress period, during stress (STR) and for five days after the last stress session (POST). There were changes in the mesor (a midline based on the distribution of values across the circadian cycles; decreased in M_2_KO by 6% over all POST), day means (inactive period of diurnal rhythm in mice; higher in M_2_KO and further increased on STR and on the second to the fifth POST) and night means (active period; lower by 13% in M_2_KO and remained decreased in STR and in POST). The total area under the curve was decreased both in the WT and M_2_KO on STR and in all POST. Repeated stress caused changes over all days of STR, but the initial values were restored in POST. The average night values were decreased, and the day means were increased by 16% over all STR in M_2_KO. The day means decreased by 14% in the 4 POST in WT. The activity biorhythm parameters were almost unchanged. We show here that stress can specifically affect heart biorhythm in M_2_KO mice, especially when the stress is acute. This implies the role of M_2_ muscarinic receptor in stress response.

## Introduction

Circadian rhythmicity, directed by the suprachiasmatic nucleus (SCN), affects the rhythmicity of local pacemakers in peripheral organs, such as the heart, liver or pancreas^1^. Each of these organs apparently has its own clockwork to regulate the transcription of genes that are important to the specific target organ. The heart, as a peripheral organ with a local pacemaker and with important adrenergic and cholinergic regulation, shows circadian rhythmicity in multiple parameters. Thus, the heart can be considered a target organ for the evaluation of circadian pacemaker effects (both central and peripheral). There are multiple indicators of heart circadian rhythmicity.

It has been shown that both β_1_-adrenoceptors (not β_2_-adrenoceptors) and basal and isoprenaline-stimulated cAMP levels change periodically over a 24 hour cycle^2^ in rat ventricular tissue. Likewise, the CREM (cAMP-inducible gene by CREB (cAMP response binding element) phosphorylation) gene has also been shown to change periodically^3^.

In contrast to rhythmic changes in adrenoceptors, cAMP and CREB, double knockout of β-adrenoceptors (β_1_/β_2_ KO mice) did not change circadian variability of heart rate (HR) and mean arterial pressure, although there were differences in activity between WT and KO animals^4^. Similarly, β_1_-adrenoceptor transgenic mice overexpressing β_1_-adrenoceptors also did not differ in circadian rhythm, although they had higher basal HR^5^. In contrast, these mice have changed HR variability (rightward shift of the high-frequency component in the dark period).

The heart is affected by civilization diseases connected with stress. Importantly, circadian variations exist in myocardial infarctions, strokes, sudden heart deaths and cardiovascular disease risk, which are associated with shift workers^6–8^. Circadian variation also exists in occurrences of myocardial ischemia, acute myocardial infarction, ventricular tachycardia, and sudden cardiac death, blood pressure, in overall disease states, and in pharmacodynamic properties^9^. Such data suggest an interconnection between stress and the heart biorhythm.

The effects of acute and chronic stress (social, psychological, yoked, escapable, unescapable) have been evaluated in multiple studies^10–20^. However, a deep biorhythmic analysis has not yet been performed, and the available studies have only reported a few parameters (mainly amplitude, i.e., night and day mean difference) changed in the stress group. Almost no data about basic chronobiology parameters, such as mesor (a midline based on the distribution of values across the cycles of the circadian rhythm, computed using a cosine function), day mean, night mean, acrophases (the time of maximal values) for different rhythm components (24-hour rhythm, 12-hour rhythm, etc.) and others are available.

In contrast, the length of the photoperiod has been shown to affect the autonomic regulation of the heart^21^. Short days increase both sympathetic and parasympathetic tone. The short day also enhanced parasympathetic withdrawal and sympathetic tone in acute short restraint stress.

The important factor in biorhythm studies is the time at which the stress was applied. However, different researchers apply stressors in different phases, or it is not possible to deduce when the stress was applied. Thus^10,13,18^, did not state when the stressor was applied^19^, and^17^ applied stressor in inactive (light) phase^15,16^, applied stressor in active phase, and^14^ used consecutive stress for 14 days. It is also necessary to emphasize that in stress studies not focused on circadian rhythmicity, the stressor is usually applied in the inactive phase, i.e., in the morning. Thus, we decided to follow the effects of the stressor applied in the inactive phase, i.e., at 8:00 AM.

It is well established that stimulation of cardiac muscarinic receptors via vagal activation triggers bradycardic responses (decreases in heart rate (HR), negative chronotropy) and that M_2_ muscarinic receptor subtype (M_2_ MR) represents the principal MR subtype found in the mammalian heart^22^. We have shown previously that despite the loss of MR agonist-induced or vagally mediated decreases in HR in M_2_ KO mice, *in vivo* basal HR^23^ in M_2_ KO mice is not changed. Moreover, an increase in heart rate was observed after carbachol in conscious M_2_ KO mice monitored telemetrically^23^. Lastly, we found that stress changes not only heart adrenoceptors but also muscarinic receptors^24–26^ and that M_2_KO are essential for signalling in the heart during restraint stress^27^. Thus, M_2_ KO mice are a suitable model for studying the role of receptor equilibrium disruption in stress processes.

To evaluate the general validity of previous results, we employed another type of stressor—restraint. Based on previous studies^16,28^, we hypothesized that prolonged stress would produce a significantly greater effect on heart biorhythm parameters than acute stress. In previous reports, it has been shown that repeated stress has more pronounced effects on different parameters (haemodynamic, heart, vascular) in experimental animals than acute stress effects. Another part of our hypothesis concerns the importance of M_2_ muscarinic receptors in stress response. We have shown repeatedly, that stress effects would be significantly affected by M_2_ muscarinic receptors^23,24,27^. Thus, we have hypothesized that M_2_ KO animals would have changes in biorhythm parameters of a greater magnitude than their WT counterparts.

## Materials and Methods

### Animals

The mouse clones lacking the M_2_ muscarinic receptor were generated in the Wess laboratory^29^ and then bred in our animal facility (Prague, Czech Republic). Animals were treated in accordance with the legislature of the Czech Republic and the EU legislature (European Convention for the Protection of Vertebrate Animals used for Experimental and other Scientific Purposes (Council of Europe No 123, Strasbourg 1985)), and the experimental protocol was approved by the Committee for the Protection of Experimental Animals of the 1^st^ Medical Faculty, Charles University, Prague and by Ministry of Education of the Czech Republic under N° MSMT-6316/2014-41. All experiments were performed in accordance with the relevant and abovementioned guidelines and regulations. The wild-type line was a mixed 129J1/CF1 line. The animals were maintained under controlled environmental conditions (12/12 light/dark cycle, 22 ± 1 °C, light on at 6:00 AM). Food and water were available *ad libitum*. Male M_2_ KO animals and their WT counterparts (weighing 20–25 g, 11–13 weeks old) were used in the study. Prior to the experiments, the mice were genotyped, and only homozygous mice were used in the study. The animals were housed three per cage to avoid social stress.

### Telemetry

**Figure 1:**
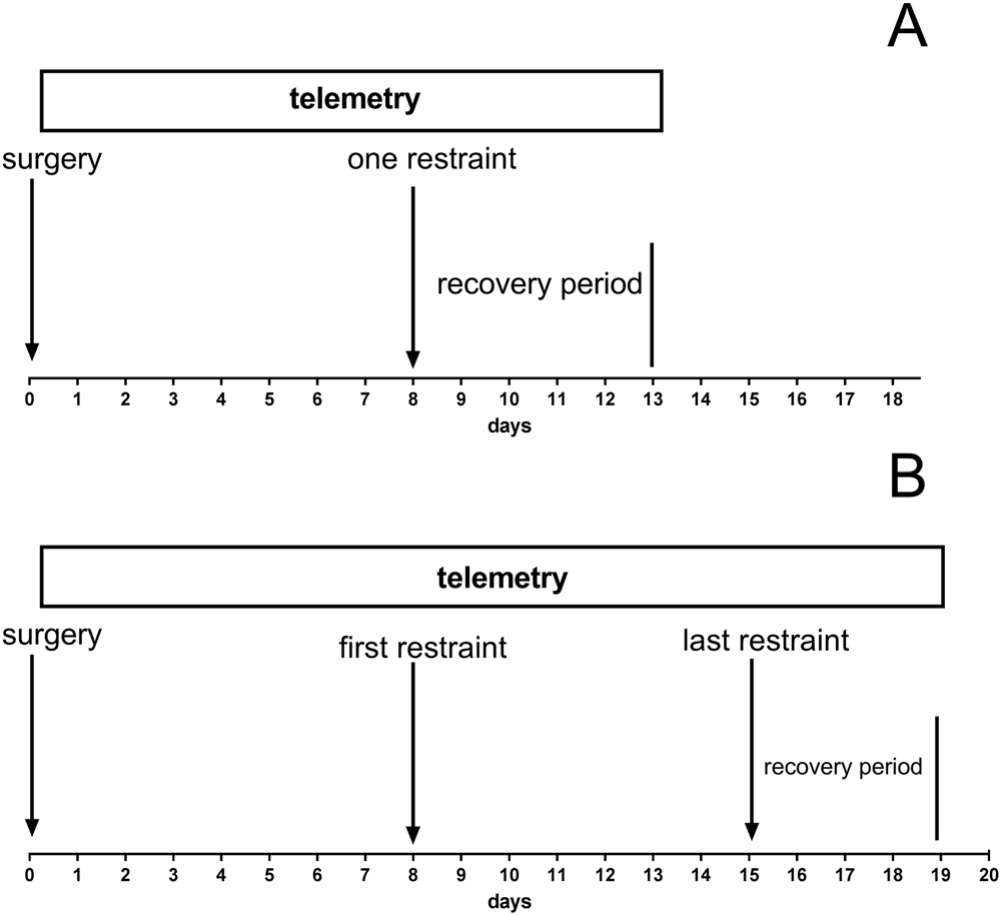
Timeline of the experiment. (**A**) Acute stress experiment. The transponders were implanted on day 0 (surgery); then, the mice were left undisturbed for one week, and on the next day (one restraint), the mice were restrained for 120 minutes. Thereafter, they were followed using telemetry for the next 5 days (recovery period). (**B**) Repeated stress experiment. The transponders were implanted on day 0 (surgery); then, the mice were left undisturbed for one week, and on the next day (first restraint), the mice were restrained for 120 minutes. This procedure was repeated the same time of the day for the next 7 days until the 15^th^ day of the experiment (last restraint). Thereafter, they were followed using telemetry for the next 5 days (recovery period).

To judge the overall functional changes in stressed animals, we employed a telemetric apparatus to measure HR and activity in intact and stressed animals and in animals in the recovery period (five days). The telemetry system is commercially available from Mini Mitter (Respironics, Andover, MA, USA, now Starr Life Sciences Corp., Oakmont, PA, USA). The transponders (E-Mitter, G2-HR) were implanted in the peritoneal cavity under the same anesthesia used in echocardiography (Zoletil® 100, Rometar® 2% 5:1, diluted 10 times, 3.2 ml.kg^−1^). The mice were left undisturbed for one week to recover from the surgery and then used in the experiment (see Fig. 1).

Sensor leads were used for HR data acquisition according to the manufacturer’s manual (i.e., in 45–60 degrees angle to transverse plane of the heart); the activity was acquired directly from the transponders. Receivers were connected in series and connected directly to the PC into a single computer port allowing for the determination of all parameters. The HR data were collected every 10 seconds, and the activity was collected every 60 seconds. VitalView was used for the initial evaluation of data.

### Restraint stress

The wild type and M_2_KO mice were exposed to acute (120 minutes, n = 20) or repeated (7 times for 120 minutes every 24 hours, n = 20) restraint sessions (see Fig. 1). The mice were placed individually in a 50 ml tube (11.5 × 3 cm) with ventilation holes, which allowed restricted movement. The stress session started at 8:00 AM, i.e., in light (non-active) period of the day in mice. The standardized transparent and perforated conic tube enables access to air and breathing, but the animal located inside is limited in its movements. Side-to-side movement is impossible, and a small extent (2 cm) of movement is possible in the forward/backward direction. The animal was placed into the tube, and within seconds, the tube was closed and fixed to the base. The control animals were not stressed and were caged in a separate room.

### Biorhythm analysis

The data collected by telemetry were grouped into half hour sequences where the mean was calculated and used for further analysis. The analysis was performed using the Chronos-fit program^30^ employing Fourier analysis and the stepwise regression technique. Then, the data were transferred into the GraphPad Prism 5.04 program (San Diego, USA) for further statistical analysis. We analyzed biorhythm parameters one week before restraint stress, during those days in which stress was applied and 5 days after the end of acute or repeated stress (i.e. in the 7 days when stress was applied and then for five consecutive days, see Fig. 1).

### Statistical analysis

Overall statistical significance was determined using Repeated measures 2-way ANOVA with Student-Neuman-Keuls post-hoc analysis and the level of stress as one factor and genotype as another factor. Statistical significance in groups was determined using Repeated measures 1-way ANOVA with Sidak post-hoc analysis. Statistical significance between groups was determined using 1-way ANOVA with Sidak post-hoc analysis. Values of p < 0.05 were considered to be significant.

## Results

### Heart rate biorhythm

#### Acute stress

**Figure 2:**
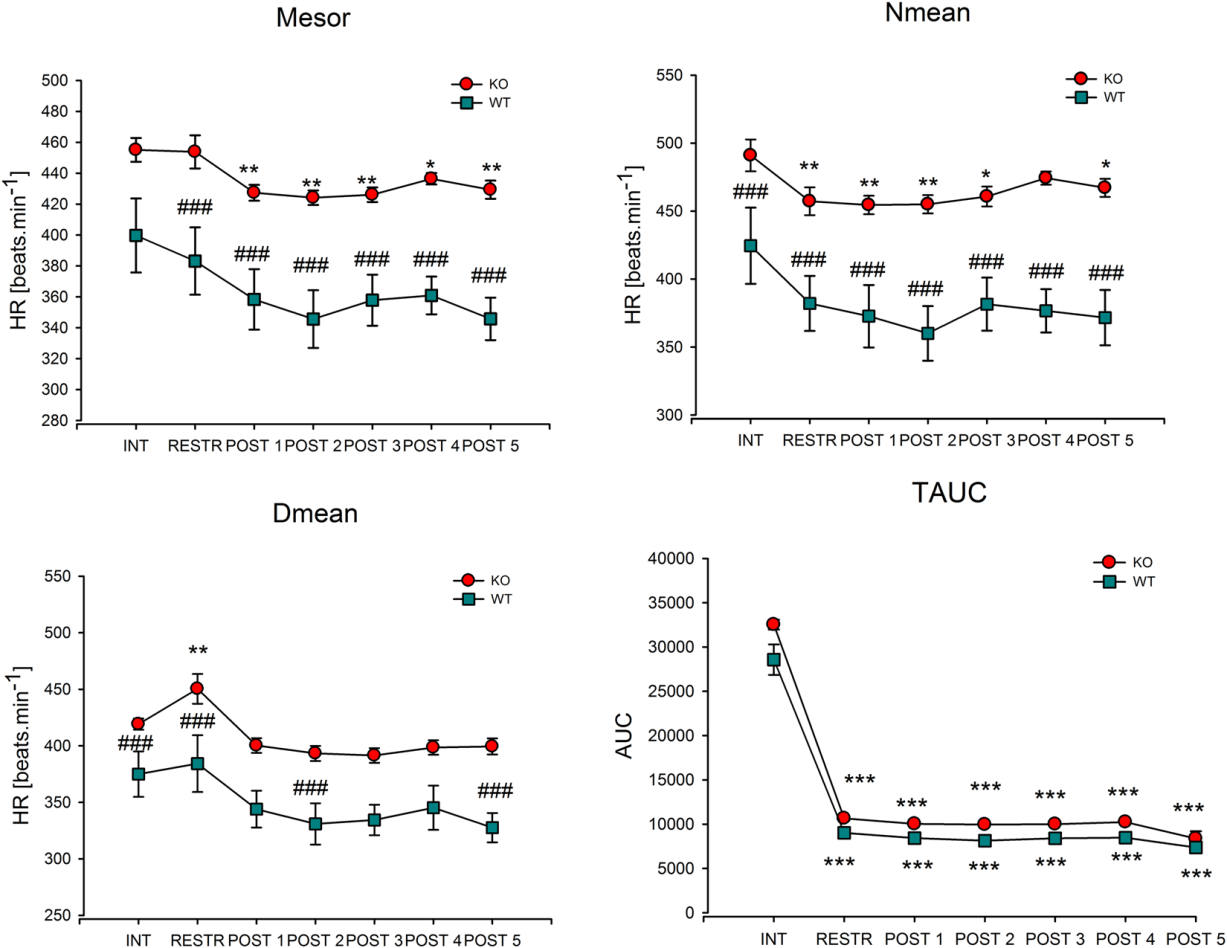
Changes in the heart rate biorhythm parameters in acute stress. Mesor, the median value in the biorhythm, Nmean, night mean, Dmean, day mean, TAUC, the total area under curve. *p < 0.05, difference from prestress period, **p < 0.01, difference from prestress period. ^###^p < 0.001, difference from WT animals. INT, intact animals, i.e., value in the prestress period (i.e., in unstressed animals), RESTR, the value during the day of restraint, POST1-POST5, the values in poststress days 1–5, HR, heart rate.

One restraint session (lasting 120 minutes) increased HR during stress similarly as has been shown recently^27^. Biorhythm analysis showed that one restraint session did not affect the mesor (a midline measure based on the distribution of values across the cycles of the circadian rhythm) in WT animals (see Fig. 2) but decreased the mesor (a midline based on the distribution of values across the cycles of the circadian rhythm) in KO animals by 6% in all poststress days. Under basal conditions, there was no difference in the mesor between WT and KO animals (see Table 1 for the main biorhythm parameter values), but it became higher in KO animals by approximately 20% during restraint (i.e., during stress) and in all poststress days. In contrast, the night mean (Nmean) was lower by 13% in KO animals and remained decreased on the day of restraint and in the following days. The day mean (Dmean) was higher in KO animals and further increased on the day of the restraint session. It was then higher on days 2 to 5 after the stress session. In KO animals, the day mean was also increased by 6% when comparing the day of the restraint session and intact animals. There was no change in night-day difference (N-D Mean, i.e., night and day mean difference that has been referred to as amplitude in previous studies) in WT or KO animals or when comparing WT to KO animals, except for the N-D Mean increase in KO animals on the day of restraint (not shown on Fig. 2). Acute stress also decreased TAUC (total area under the curve representing the area between the x-axis and the fitted curve) both in WT and KO animals on the day of restraint and in all days following the stress session. Other biorhythm parameters did not differ in the stress group when compared to intact animals.

**Table 1:** Rhythm analyses of HR (Heart rate), and ACT (motor activity) in WT and M_2_ KO mice in basal condition, i.e. in intact animals.

	WT				M_2_ KO			
	HR		ACT		HR		ACT	
	Mean	SEM	Mean	SEM	Mean	SEM	Mean	SEM
Mesor	430.14	20.96	10.14	0.04	466.93	11.35	10.19	0.62
Day	406.11	18.26	6.67	0.30	428.80	10.13	5.89	0.37
Night	454.17	24.05	13.60	0.75	505.07	13.13	14.50	0.94
N-D	48.06	8.16	6.93	0.74	76.27	5.87	8.61	0.68

The mesor, nighttime (Night) and daytime (Day) mean values, including the night-day difference (N-D), are shown. Data were analyzed using one-way ANOVA with Sidak’s test used for post-hoc analysis. Data are expressed as mean ± SEM. No differences were found on these basal biorhythm parameters.

#### Repeated stress

**Figure 3:**
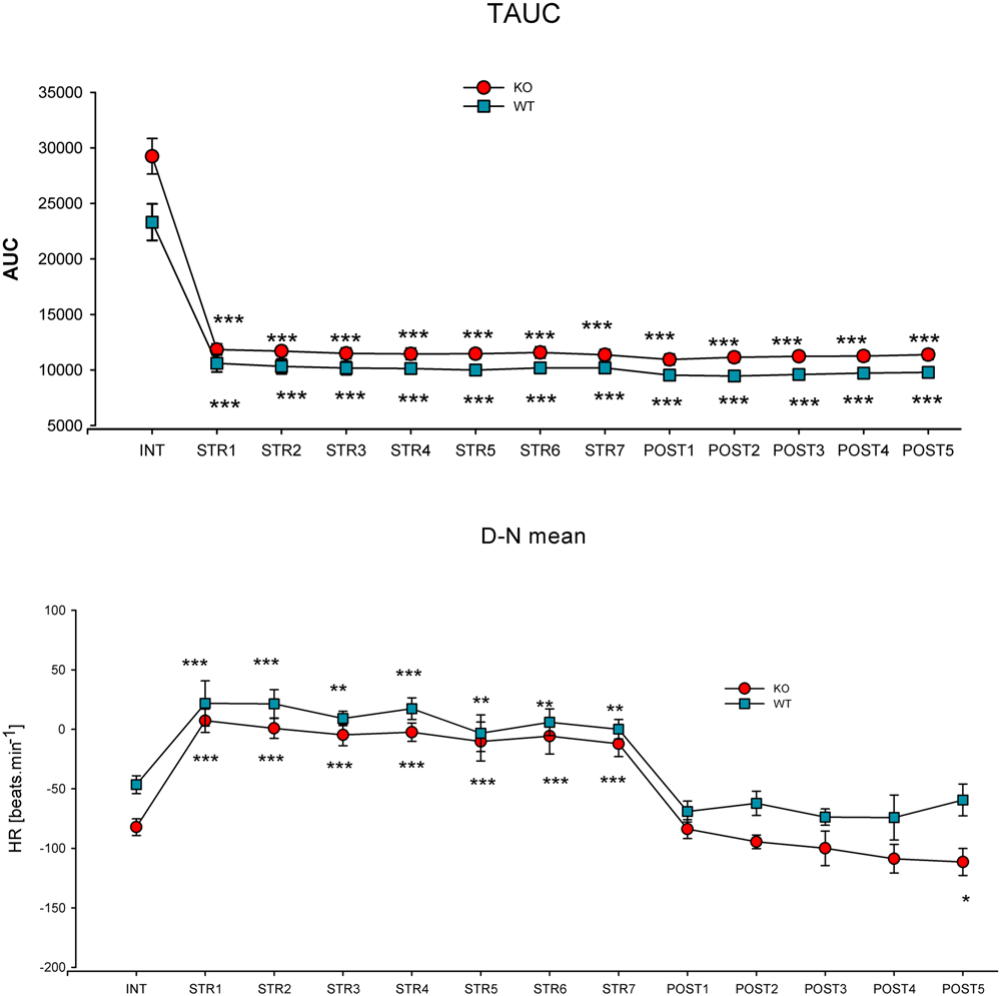
Changes in the total area under curve (TAUC, top) and difference between day and night means (D-N mean, bottom) in the repeated stress condition. *p < 0.05, difference from prestress period, **p < 0.01, difference from prestress period (i.e., in unstressed animals), ***p < 0.001, difference from prestress period. INT, intact animals, i.e., value in the prestress period, STR 1–7, the value during the first to seventh day of restraint, POST1-POST5, the values in poststress days 1–5, HR, heart rate.

**Figure 4:**
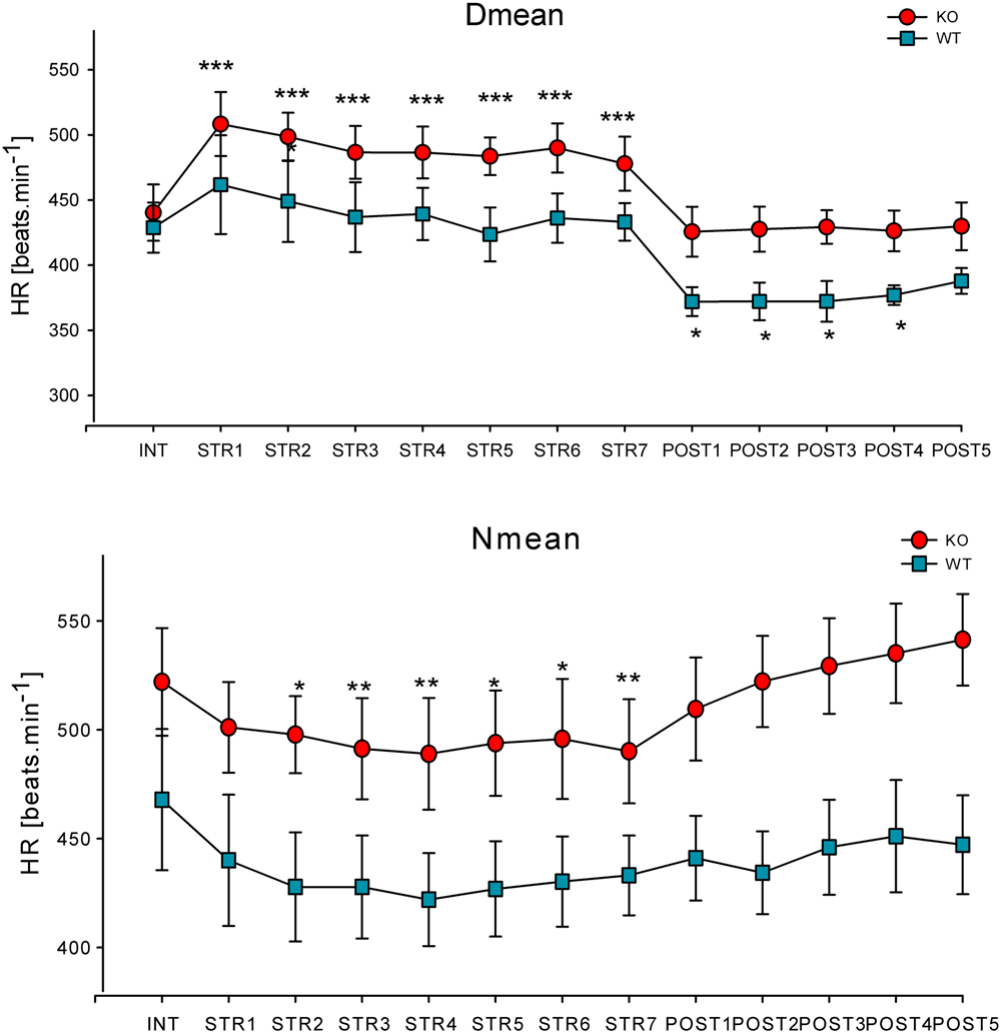
Changes in the day mean (Dmean, top) and the night mean (Nmean, bottom) in the repeated stress conditions. *p < 0.05, difference from prestress period, **p < 0.01, difference from prestress period, ***p < 0.001, difference from prestress period. INT, intact animals, i.e., value in the prestress period (i.e., in unstressed animals), STR 1–7, the value during the first to seventh day of restraint, POST1-POST5, the values in poststress days 1–5, HR, heart rate.

It is evident that repeated stress caused changes in biorhythm parameters in all days when animals were restrained. However, the animals revealed a very good restoration ability to return to initial values in the after-stress period. There was an exception in TAUC (see Fig. 3), where values remained decreased in all five days after the stress in both WT and KO animals. The D-N mean (see Fig. 3) was decreased on the fifth day after stress in KO animals. The Tmean (total mean of all measured values) was decreased in WT animals on the second day after stress (not shown). It is interesting that the Nmean (see Fig. 4), i.e., the average values in the night (when stress was applied in the light period) were decreased in KO animals, while it was not changed in WT animals. After the end of stress, there were no changes in either genotype. Interestingly, the Dmean (see Fig. 4) was unchanged during the stress period in WT animals (but increased by 16% in KO animals on all days when stress was applied). After the stress exposure, it decreased by 14% in the 4 poststress days in WT animals. The lowest night value (N lowest, not shown) was increased on the fifth day after stress in KO animals; in WT animals, there was a small decrease on the 3^rd^ day of restraint. Other biorhythm parameters did not differ in the stress group when compared to intact animals.

### Activity

#### Acute and repeated stress

In total contrast to HR, the activity biorhythm parameters were changed only moderately. There were changes in TAUC in acute and repeated stress only (not shown on figure). In addition, there was also a decrease in plengths (number of periods) on day 5 after acute stress. No other changes were revealed.

## Discussion

Here, we show that acute stress affects heart biorhythm parameters and that these changes can be seen mainly when the main cardioinhibitory receptors—M_2_ MR—are lacking. WT animals were relatively resistant to stress-induced biorhythm changes. When the stress influences the animal repeatedly, the animals can cope with such changes. Previously, we^23,27^ showed that HR is increased during stress and decreases afterwards and that there are HR differences in the dark and light periods. This can explain the present results regarding unchanged HR (Dmean, i.e., in the inactive (light) phase of the day). Almost no changes were observed in the activity biorhythm, suggesting that the HR biorhythm changes were specific. Thus, we can conclude that M_2_ MR plays an important role in coping with stress.

As stated above, the effects of stress on biorhythm parameters have not been systematically investigated. Although^28^ have investigated the effects of stress on HR and mean arterial pressure using a telemetry system, the analyses of biorhythms were not performed. They found an increase in HR during the stress sessions, which was also the case in this study, and similar to our study, they also observe a robust ability to restore the initial values after the end of the stress period (they applied the stressor six times). Social stress (confrontation of intruder and resident) was able to decrease the amplitude of circadian rhythm in the intruder (HR and core temperature) when repeated five times^31^. The effects of chronic social stress (everyday changes in the social group of males housed with the females) have been studied in normotensive and borderline hypertensive rats^10^ for one month, and no changes have been reported in HR and blood pressure in normotensive and borderline hypertensive rats. However, these authors did not provide an extensive analysis of the biorhythm. Nevertheless, these results are in partial agreement with our data regarding the relatively good adaptability of HR biorhythm to chronic stress. Acute surgery and social stress (defeat) were studied by^13^. Surgery had a more profound effect of circadian parameters, as the circadian amplitude of heart rate and temperature increased significantly nine days after surgery. Circadian temperature amplitude increased following social defeat only. Acrophase of temperature but not heart rate changed significantly after surgery but not after the social defeat. Moreover, additional ultradian rhythms were revealed after stress. The HR mesor, however, did not differ. This is in variance with our data, but we employed a different type of stressor (restraint). Long-term consequences of social conflict in rats have been studied by^15^. The authors found decreases in HR, temperature and activity amplitude. No reference is given about mesor changes. In further study from this laboratory^19^, found that repeated (ten episodes) social defeat stress produced a decrease in the HR amplitude and only minor changes in the daily rhythms of body temperature and physical activity. Although these authors paid attention to various heart parameters (HR variability, R-R intervals), the biorhythm parameters reported in that paper were limited to changes in HR, temperature and activity amplitude. Chronic (14 days) sound stress^14^ did not cause changes in the mean and amplitude of HR and temperature. The heart rate, motor activity and body temperature of freely moving male mice were compared between mice housed either individually or in pairs with an ovarectomized female^12^. HR was increased in mice housed individually, temperature was reduced, and activity did not differ. Conversely, miniature swine^11^ revealed an HR increase when pair housed in comparison to single housing. Although the HR biorhythm was described, these authors also did not deeply analyze the biorhythm. A one-hour foot shock session over five consecutive days^18^ decreased HR both in the active and inactive periods and decreased activity in the active (dark) period. However, again, no further biorhythm analysis was performed. Uncontrollable (unescapable) and controllable (escapable) stress (tail shock) effects on HR, activity and temperature biorhythm were compared in the study of^17^. Both types of stress (controllable and uncontrollable) disrupted diurnal rhythms of locomotor activity and body temperature but not heart rate. Similarly, as in previously mentioned studies, biorhythm analysis was not performed. Diurnal variation in hemodynamic parameters (again without further biorhythm analysis) was demonstrated after restraint stress^16^.

The Lemmer’s group^32^ studied the changes in HR and blood pressure (systolic, diastolic, mean) after short-term restraint stress (25 minutes, followed by another 30 minutes) in WKY and SHR rats and did not find differences between these strains. It was thus concluded that stress increases HR, which has also been shown in humans when exposed to a combination of psychological and physical stress as reviewed by^33^.

In contrast to our hypothesis, acute stress had more profound effects on HR biorhythm than repeated stress. However, the stress used by us (restraint) was not as strong as the immobilization of whole body by taping it to a fixed board in the prone position^34^. Despite this, acute stress has a different pattern of hormonal changes than repeated stress (see, e.g.,^35–38^). Additionally, hormone levels correlated with cardiovascular responses in humans^39^. Thus, different hormone levels can be one of the determining factors of different biorhythm responses to acute and repeated stress.

We have shown previously that heart biorhythm parameters (*in vivo* basal HR) display only marginal differences between WT and M_2_ KO animals^23^. However, acute stress can change multiple parameters in animals, especially when lacking the M_2_ MR. Thus, the susceptibility to stress can be affected by a normal balance between cardiostimulatory and cardioinhibitory receptors, i.e., between β-adrenoceptors and MR, and this balance is disrupted in M_2_KO animals. Susceptibility to stress (induced by isoprenaline bolus) has also been reported in M_2_ KO^40^. Similarly, our previous finding showed differences in drug response (carbachol, isoprenaline, atropine, but not propranolol) in nonstressed animals^23^. Recently, we have also shown that changes in M_2_ MR signaling during stress are modified in M_2_ KO^27^.

We can conclude that stress can specifically affect the heart biorhythm. These effects are more pronounced in acute (one session) stress than in repeated (seven sessions) stress. As demonstrated previously, M_2_ KO mice are more susceptible to stress, and thus, these effects are more highly expressed in M_2_KO animals. This implies the role of M_2_ muscarinic receptor in stress response. We can conclude that not only muscarinic receptor density, activated pathways, heart rate and agonists/antagonists effects on heart rate are affected by lack of M_2_ muscarinic receptor, but also the heart rate biorhythm is affected when these receptors are missing. Very probably, the peripheral pacemaker is responsible for this biorhythm changes, as the activity biorhythm was almost unaffected by lack of M_2_ muscarinic receptor.
